# Determinants of HIV voluntary counseling and testing: a multilevel modelling of the Ethiopian Demographic and Health Survey

**DOI:** 10.1186/s12905-021-01590-0

**Published:** 2022-01-08

**Authors:** Adugnaw Zeleke Alem, Achamyeleh Birhanu Teshale, Alemneh Mekuriaw Liyew, Getayeneh Antehunegn Tesema, Ayenew Kassie Tesema, Yigizie Yeshaw

**Affiliations:** 1 Department of Epidemiology and Biostatistics, Institute of Public Health, College of Medicine and Health Sciences, University of Gondar, Gondar, Ethiopia; 2 Department of Health Education and Behavioral Science, Institute of Public Health, College of Medicine and Health Sciences, University of Gondar, Gondar, Ethiopia; 3 Department of Physiology, School of Medicine, College of Medicine and Health Sciences, University of Gondar, Gondar, Ethiopia

**Keywords:** VCT uptake, Reproductive age women, HIV/AIDS, Ethiopia

## Abstract

**Background:**

Human immunodeficiency virus (HIV) counseling and testing services are vital to reduce the spread of HIV infection, and to create an opportunity for early treatment and reduction of HIV/AIDS-related mortality. However, only 12 sub-Saharan African (SSA) countries reached the first 90% target (90% of people living with HIV to know their status). Hence, this study aimed to investigate the determinants of HIV counseling and testing among reproductive-age women in Ethiopia.

**Methods:**

Ethiopian Demographic and Health Survey (EDHS 2016) data was used to identify the determinants of HIV counseling and testing among reproductive-age women in Ethiopia. A weighted sample of 14,599 reproductive age women was included in the study. A multilevel binary logistic regression model was fitted to identify the determinants of HIV counseling and testing. The odds’ ratio with a 95% Confidence Interval (CI) and the corresponding P-value ≤ 0.05 was employed to declare the statistically significant variables.

**Results:**

In this study, both individual and community-level variables were significantly associated with Voluntary Counseling and Testing (VCT) uptake among women. Women aged 25–34 years (Adjusted Odds Ratio (AOR) 2.29, 95% CI 2.05, 2.56), aged ≥ 35 years (AOR 1.55, 95% CI 1.38, 1.75), attending primary education (AOR 1.68, 95% CI 1.51, 1.88), secondary education (AOR 3.07, 95% CI 2.64, 3.58), and higher education (AOR 5.15, 95% CI 4.17, 6.36), women with medium household wealth (AOR 1.56, 95% CI 1.32, 1.84), richer (AOR 1.88, 95% CI 1.58, 2.24), and richest wealth index (AOR 2.37, 95% CI 1.91, 2.94), having comprehensive knowledge (AOR 1.21, 95% CI 1.06, 1.37), ever married (AOR 3.87, 95% CI 3.46, 4.32), having sexual risky behavior (AOR 2.09, 95% CI 1.69, 2.49), women from communities with high HIV knowledge (AOR 2.03, 95% CI 1.68, 2.45), women from communities with high literacy level (AOR 1.16, 95% CI 1.05, 1.51) and women from communities with high wealth quintile (AOR 1.20, 95% CI 1.03,1.57) had higher odds of VCT uptake. However, those women having stigma (AOR 0.81, 95% CI 0.74, 0.92) had reduced odds of VCT uptake.

**Conclusion:**

This study revealed that not only individual level factors but also community level factors determine the status of HIV voluntary counseling and testing. Hence, strengthening both individual and community based interventions are crucial to increase the women HIV counseling and testing practice in the country.

## Background

Human Immunodeficiency Virus (HIV) is a major public health problem worldwide, with sub-Saharan Africa being the most affected region [1, 2]. In 2017, 36.9 million people worldwide were living with HIV, in which 50% (19.6 million) were living in Eastern and Southern Africa [2]. The prevalence of HIV in Ethiopia is 0.9% in 2016 [3].

In 2014, the United Nations Program on HIV/AIDS (UNAIDS) has adopted the 90–90–90 strategic framework [4]. This framework calls for 90% of People Living with HIV (PLWHA) to know their status, 90% of those diagnosed receiving treatment, and 90% of those receiving treatment being virally suppressed by 2020 [5]. In line with this program, our country, Ethiopia, started Voluntary Counseling and Testing (VCT) for the larger community after the national HIV/AIDS policy was launched [5] and a remarkable achievement was recorded on the last two strategies of UNAIDS. However, only 79% of PLWHA are aware of their status [6]. In order to end HIV by 2030, the focus of global efforts is to increase VCT to reduce and eliminate new infections in key populations [7]. However, the global trend of HIV infection increases due to high proportions of PLWHA who are still unaware of their status [2, 8]. Although proportion of PLWHA who know their HIV status increased from 54% in 2015 to 84% in 2020 globally, only 12 sub-Saharan African (SSA) countries reached the first 90% target, without which the control of the epidemic will not be achieved [9–11]. In Africa, VCT coverage ranges from 7.6% in Madagascar to 85.7% in Rwanda [12–15]. The Federal Democratic Republic of Ethiopia has committed to reducing new adult HIV infections by 50 percent by 2020 and to ending AIDS as a public health threat by 2030 [16]. Hence, VCT is a critical step to reduce the risk of spreading HIV, to access early HIV-specific treatment, care and support, to manage one’s health, and to plan for the future for the benefit of themselves and others. Despite these advantages of VCT and Ethiopian government provides free VCT service, less than half of reproductive age women receive HIV testing services in Ethiopia [12, 17–19].

Different determinants have been associated with VCT uptake among women. These include: education status of women [1, 20–24], marital status [18, 25–27], wealth status [18, 19, 22–24, 27], age [18, 19, 22, 23, 25, 26, 28], sexual behavior [18, 19, 22, 24, 26], religion [18, 19, 24], residence [19], knowledge related to HIV [18, 21, 23–26, 28], stigma [18, 19, 21–23, 27], antenatal care visit [28, 29], knowledge on maternal to child transmission [28], occupation [20, 24], distance from health facility [22], know where to get VCT [27], media exposure and number of children [24].

Although community-owned interventions are important to increase HIV testing and counseling for women, the country’s limited research in this area only focuses on the individual determinants of VCT, leaving community-level determinants behind. Moreover, almost all of them are conducted on some parts of the country, not nationally representative. Therefore, this study aimed to determine both individual and community level determinants of VCT uptake among reproductive-age women in Ethiopia using multilevel analysis, a model that accounts for the hierarchical nature of Ethiopian Demographic and Health Survey (EDHS) data.

## Methods

### Data source and sampling technique

**Fig. 1:**
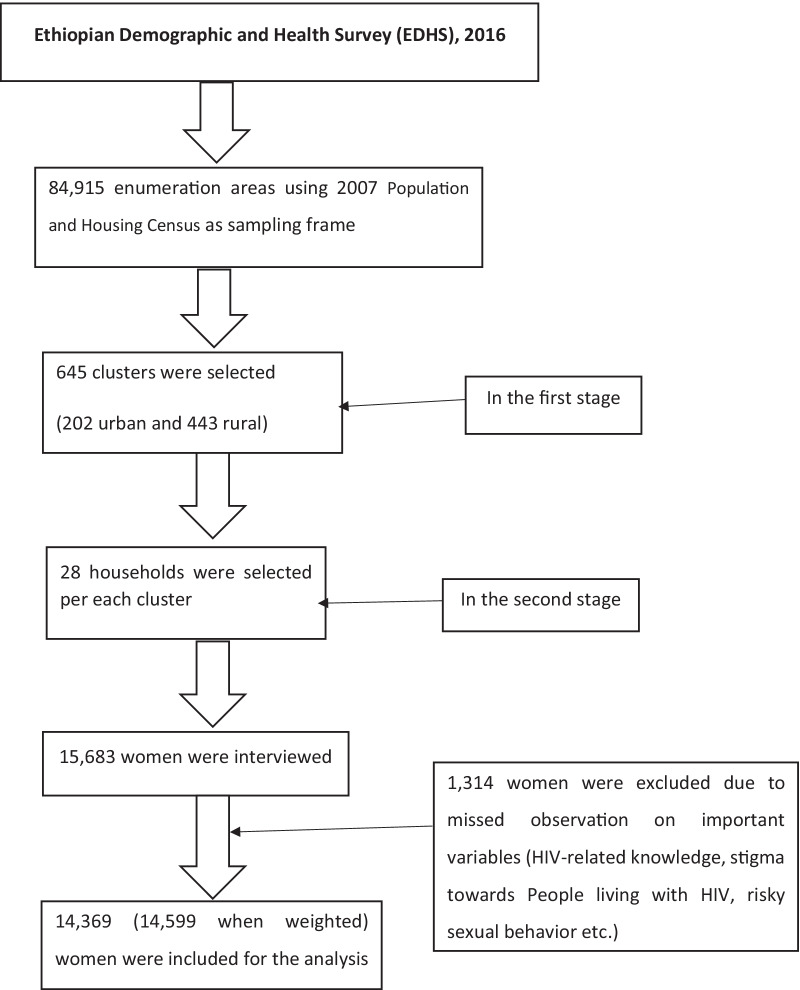
Flowchart of Ethiopian Demographic and Health Survey sampling procedure and data extraction

We used Ethiopian Demographic Health Survey (EDHS) 2016 data, a recent population-based survey. The Ethiopian Demographic Health Survey was designed to provide population and health indicators at the national and regional levels. The 2016 EDHS used a stratified two-stage cluster sampling procedure. In the first stage of the sampling, 645 clusters (202 urban and 443 rural) were selected on the basis of the 2007 Ethiopian population and housing census sampling frame using systematic sampling with probability proportional to size. In the second stage, a fixed number of 28 households per cluster were selected randomly from the household listing. Data were collected using a structured interviewer-administered questionnaire. To ensure data quality questionnaires were pretested, training was given for both data collectors and supervisors. A women’s questionnaire (IR file) which collected information from all eligible women aged 15–49 years was used for this study. These women were asked questions on the following topics: background characteristics, birth history and childhood mortality, family planning, fertility preferences, and adult and maternal mortality [30]. In this study, aweighted sample of 14,599 reproductive age women were included (Fig. 1).

### Study variables

The outcome variable for this study was VCT uptake, which was determined by asking a question, ‘have you ever tested for HIV? The responses of the participants were coded as 0 (No) and 1 (Yes). The independent variables for this study were classified as individual and community level determinants. Individual-level variables were: women’s education, age of respondents, number of ANC visits, religion, working status, media exposure, wealth index, HIV/AIDS-related knowledge, HIV/AIDS-related stigma towards PLWHA and risky sexual behavior. HIV/AIDS knowledge was assessed by using six questions included in the EDHS questionnaires, which are related to HIV prevention and misconceptions. The score was obtained by giving one point to respondents who knew the correct response and 0 to those who answered the incorrect response. HIV related knowledge was categorized into three categories as having low knowledge (score ≤ 3), high knowledge (score 4–5) and comprehensive knowledge (score 6) knowledge. Stigma towards PLWHA was assessed using a set of six questions, and it was categorized as “no stigma” (score 6), “low stigma” (score 4–5), “moderate stigma” (score 2–3), and “high stigma” (score ≤ 1). Risky sexual behavior was measured using a set of five questions and categorized as “no risk” (score 0), “some risk” (score 1) and “high risk” (score ≥ 2).

Community-level variables include: residence, perception of distance from health facility, community literacy level of women (aggregate value of the individual level factor women’s educational level by considering the proportion of women who completed primary and above educational level in the primary sampling unit), community wealth level (computed from the household wealth by considering proportion of women in the community that are from the poorest and poorer household wealth), community knowledge (proportion of women in the community that have at least high HIV related knowledge), community media exposure (proportion of women in the community who had exposure to at least one type of media; radio, newspaper, internet or television), HIV-related stigma (percentage of respondents in the community with stigma towards PLWHA) and sexual risk behavior (proportion of women in the community that have HIV risk perception). Community-level determinants were obtained by aggregating individual responses for each item to the community (cluster) level based on the women in the sample. We used the national median as a cut-off point to divide each community variable into low and high.

### Statistical analysis

The data were checked for completeness and weighted before doing any statistical analysis. A sampling weight was done during computation of the descriptive statistics to adjust for the non-proportional allocation of the sample to different regions and their urban and rural areas. The data were analyzed using Stata 14 software. Four models were constructed for the data. We first performed a null model (a model with no predictor), then model I (a model with the individual-level variables) and model II (a model with community-level variables). Finally, model III (a model that includes both individual and community level variables) was constructed. Deviance was used to select the appropriate model. Therefore, model III (model with the lowest deviance) was chosen to interpret all results.

Both bivariable and multivariable multilevel logistic regression model were used to identify determinants of VCT uptake. In the bivariable multilevel logistic model analysis, all variables with p < 0.2 were input into the final model. In this model, odds ratio with 95% Confidence Interval (CI) was estimated for the potential determinants of VCT uptake among women. P-value ≤ 0.05 was employed to declare the statistically significant variables.

## Results

### Background characteristics of respondents

In this study, a weighted sample of 14,599 reproductive age women were included. Of these, 2448 (70.2%) of women ever tested HIV were living in urban. Forty percent of women ever tested has no education, but the majority (82.1%) of ever tested were attended higher education. Three-quarters of study participants who had been tested for HIV reported that they had ≥ 4 ANC follow ups. Sixty-eight percent of women from the richest households and 28.7% of the poorest households have been tested for HIV. Approximately two-thirds (63.2%) of women who have been tested have a comprehensive knowledge of HIV prevention and misconceptions. More than half (53.7%) women living in communities where the proportion of respondents were more educated than the median were ever tested (Table 1).

**Table 1:** Frequency distribution of VCT utilization among reproductive-age women in Ethiopia, 2016

Variables	HIV testing and counseling	
	Tested for HIV (%)	Not tested for HIV (%)
*Residence*		
Rural	4482 (40.1)	6680 (59.9)
Urban	2448 (70.2)	989 (29.8)
*Religion*		
Muslim	1665 (38.9)	2612 (61.1)
Orthodox	3764 (57.6)	2769 (42.4)
Protestant	1408 (40.3)	2086 (59.7)
Catholic	41 (36.3)	72 (63.7)
Others	50 (28.1)	128 (71.9)
*Marital status*		
Never married	2146 (40.3)	3182 (59.7)
Ever married	4785 (51.6)	4486 (48.4)
*Women’s education*		
No education	2657 (40.2)	3958 (59.8)
Primary	2417 (45.7)	2868 (54.3)
Secondary	1120 (62.0)	685 (38.0)
Higher	718 (82.1)	157 (17.9)
*Wealth Index*		
Poorest	642 (28.7)	1593 (71.3)
Poorer	935 (37.1)	1584 (62.9)
Middle	1102 (39.9)	1658 (60.1)
Richer	1452 (48.9)	1516 (51.1)
Richest	2798 (68.0)	1316 (32.0)
Media exposure		
No	3165 (39.7)	4806 (60.3)
Yes	3765 (56.8)	2863 (43.2)
*Knowledge*		
Low	1420 (30.9)	3176 (69.1)
High	3678 (51.8)	3428 (48.2)
Comprehensive	1831 (63.2)	1065 (36.8)
*Stigma*		
No	387 (61.3)	244 (38.7)
Low	2291 (53.9)	1957 (46.1)
Moderate	2971 (48.9)	3110 (51.1)
High	1280 (35.2)	2356 (64.8)
*Sexual risky behavior*		
No	6398 (46.4)	7396 (53.6)
Some	461 (66.7)	230 (33.3)
High	70 (62.5)	42 (31.5)
*Number of ANC*		
0	597 (24.6)	1831 (75.4)
1	145 (48.5)	154 (51.5)
2	272 (48.3)	291 (51.7)
3	836 (63.5)	481 (35.5)
≥ 4	1784 (75.6)	571 (24.4)
*Distance to health facility*		
Big problem	2594 (36.4)	4535 (63.6)
Not Big problem	4335 (58.0)	3133 (42.0)
*Community-level literacy*		
Low	1418 (32.7)	2916 (67.3)
High	5513 (53.7)	4752 (46.3)
*Community wealth*		
Low	2513 (35.3)	4604 (64.7)
High	4417 (59.0)	3065 (41.0)
*Community media exposure*		
Low	3082 (36.8)	5385 (63.2)
High	3848 (62.7)	2284 (37.3)
*Community knowledge*		
Low	1464 (31.7)	3160 (68.3)
High	5467 (54.8)	4508 (45.2)
*Community stigma*		
Low	3567 (57.4)	2652 (32.6)
High	3363 (40.1)	5017 (59.9)

#### HIV*/AIDS-related knowledge, stigma and risky sexual behavior*

Fifty-four percent of respondents who were tested for HIV knew that regular use of condoms during sex can reduce the risk of HIV infection. More than half (52.2%) of respondents who had been tested for HIV believed that HIV infection was not transmitted by mosquito bites. The majority (59.4%) of participants tested for HIV believed that children should be allowed to go to school with. The majority (59.4%) of participants who had been tested for HIV believed that HIV-infected children HIV should be allowed to go to school r with HIV-uninfected children. Forty-two percent of women who had multiple sexual partners and two-third of women who had any STI in the last 12 months had ever been tested for HIV (Table 2)*.*

**Table 2:** VCT uptake in relation to HIV/AIDS-related knowledge, HIV/AIDS-related stigma and risky sexual behavior history among Women in Ethiopia, 2016

Variables	Tested for HIV (%)	Not tested for HIV (%)
*Knowledge indicators*		
Always use condoms during sex (Yes)	4909 (54.2)	4140 (45.8)
Have 1 sex partner only, who has no other partners (Yes)	5429 (50.2)	5391 (49.8)
Can get HIV from mosquito bites (No)	3970 (52.2)	3631 (47.8)
Can get HIV by sharing food with person who has AIDS(No)	6047 (52.6)	5456 (47.8)
Can get HIV by witchcraft or supernatural means (No)	5974 (51.5)	5636 (48.5)
A healthy looking person can have HIV (Yes)	4995 (52.9)	4446 (47.1)
*Stigma indicators*		
Would be ashamed if someone in the family had HIV (Disagree)	4320 (54.0)	3682 (46.0)
Would buy vegetables from vendor with HIV (Yes)	3761 (59.1)	2593 (40.9)
Children with HIV should be allowed to attend school with children without HIV (Yes)	4272 (59.4)	2915 (40.6)
People hesitate to take HIV test because reaction of other people if positive (No)	1717 (42.1)	2366 (51.9)
People talk badly about people with or believed to have HIV (No)	3555 (52.2)	3249 (47.8)
People with or believed to have HIV lose respect from other people (No)	3884 (52.1)	3575 (47.9)
*Risky sexual behavior indicators*		
Had any STI in last 12 months (Yes)	28 (66.7)	14 (33.3)
Had genital sore/ulcer in last 12 months (Yes)	133 (55.4)	107 (44.6)
Had genital discharge in last 12 months (Yes)	169 (58.9)	118 (41.1)
At least one sexual partner other than husband in last 12 months(Yes)	5429 (50.2)	5391 (49.8))
Had multiple life time sexual partner (Yes)	2601 (42.2)	3531 (57.8)

### Random effect analysis

The results of the random-effects model indicated that there was a significant clustering of VCT uptake across the communities. The intra-class correlation (ICC) in the empty model indicates that 33% of the overall variability in VCT uptake is attributed to cluster variability. The median odds’ ratio for VCT uptake was 3.36 in the empty model, which indicates that there was variation between clusters. This showed that if we randomly select women from different clusters, women at the cluster with higher VCT uptake had 3.36 times higher odds of having VCT uptake as compared with women at cluster with lower VCT uptake. As shown by Proportional Change in Variance (PCV), 63% of the variability in VCT uptake was explained by the full model with both individual and community level variables (Table 3).

**Table 3:** Random effect and model fitness of VCT uptake among reproductive-age women

Random measures	Null model	Model I	Model II	Model III
Community-level variance	1.63	0.79	0. 64	0.61
ICC	0.33	0.19	0.16	0.15
MOR	3.36	2.33	2.14	2.10
PCV (%)	Reference	52	61	63
*Model fitness*				
DIC	17,533.50	15,637.31	17,064.92	15,477.80

DIC, Deviance Information Criterion; ICC, Intraclass Correlation Coefficient; MOR, Median Odds Ratio; PCV, Percentage Change in Variance

### Determinant of VCT uptake among women of reproductive age

#### Fixed effect

As mentioned in the method section above, first we have the empty model, and then we insert the individual level determinants, and we get odds ratios, thirdly, we insert the community level determinants, and we calculated the adjusted odds ratios and at last we insert the individual and community level determinants combined and get another odds’ ratio with their corresponding 95% CI. Among the four models fitted, the model with individual and community-level determinants has the lowest deviance and is therefore considered to be the best fitting model. Accordingly, educational status, age, wealth index, marital status, HIV related knowledge, sex risky behavior, stigma, community wealth, community-level literacy and community HIV related knowledge were significantly associated with VCT uptake among women of reproductive age (p ≤ 0.05).

There is a significant positive association between education level and VCT acceptance. The odds of VCT uptake among women with primary, secondary and higher education were 1.68 (95% CI 1.51, 1.88), 3.07 (95% CI 2.64, 3.58) and 5.15 (95% CI 4.17, 6.36) times higher than women with no education, respectively. This study shows that the use of VCT increases as household wealth increases. Women from Richest (Adjusted Odds Ratio (AOR) 2.37, 95% CI 1.91, 2.94), richer (AOR 1.88, 95% CI 1.58, 2.24), medium (AOR 1.56, 95% CI 1.32, 1.84) and poorer households (AOR 1.46, 95% CI 1.25, 1.70) had higher odds of VCT uptake as compared with women from households with the poorest wealth status. Enabling factors such as women who have comprehensive knowledge related HIV prevention and misconception had higher odds of VCT uptake (AOR 1.21, 95% CI 1.06, 1.37) compared to women with low knowledge. Compared with women aged 15–24 years, women aged 25–34 years and ≥ 35 years had higher odds of VCT uptake (AOR 2.29, 95% CI 2.05, 2.56), (AOR 1.55, 95% CI 1.38, 1.75), respectively. Additionally, VCT uptake was significantly higher among ever married (AOR 3.87, 95% CI 3.46, 4.32), and having sexual risky behavior (AOR 2.09, 95% CI 1.69, 2.49) compared to never married and women with no sexual risk behavior, respectively. Women with highly stigmatizing views of PLWHA had lower odds of VCT uptake (AOR 0.81, 95% CI 0.74, 0.92) than women without stigma.

The odds of VCT uptake among women from communities with high HIV-related knowledge was 2.03 (AOR 2.03, 95% CI 1.68, 2.45) times higher compared to the communities with low HIV related knowledge. The odds of VCT uptake was higher among women living in communities with a higher percentage of education (AOR 1.16, 95% CI 1.05, 1.51) and women from communities with high wealth quintiles (AOR 1.20, 95% CI 1.03, 1.57) compared to their counterparts (Table 4).

**Table 4:** Multilevel logistic regression analysis for factors associated with VCT uptake among Ethiopian women, 2016

Variables	Model I AOR (95% CI)	Model II AOR (95% CI)	Model III AOR (95% CI)
*Age*			
15–24	1		1
25–34	2.34 (2.10–2.61)		2.29 (2.05–2.56)
≥ 35	1.16 (1.43–1.82)		1.55 (1.38–1.75)
*Marital status*			
Never married	1		1
Ever married	3.71 (3.32–4.15)		3.87 (3.46–4.32)
*Women’s education*			
No education	1		1
Primary	1.76 (1.58–1.97)		1.68 (1.51–1.88)
Secondary	3.32 (2.85–3.86)		3.07 (2.64–3.58)
Higher	5.57 (4.53–6.89)		5.15 (4.17–6.36)
*Working status*			
Not working	1		1
Working	1.32 (1.20–1.44)		1.28 (0.98–1.40)
*Wealth index*			
Poorest	1		1
Poorer	1.63 (1.40–1.89)		1.46 (1.25–1.70)
Middle	1.85 (1.58–2.17)		1.56 (1.32–1.84)
Richer	2.37 (2.01–2.79)		1.88 (1.58–2.24)
Richest	4.24 (3.58–5.06)		2.37 (1.91–2.94)
*Knowledge*			
Low	1		1
High	1.15 (1.04–1.28)		1.07 (0.97–1.19)
Comprehensive	1.34 (1.18–1.56)		1.21 (1.06–1.37)
*Stigma*			
No	1		1
Low	0.97 (0.80–1.18)		0.99 (0.81–1.20)
Moderate	0.91 (0.75–1.10)		0.96 (0.79–1.17)
High	0.82 (0.66–1.01)		0.81 (0.74–0.92)
*Media exposure*			
No	1		1
Yes	1.09 (0.99–1.21)		1.01 (0.90–1.11)
*Sexual Risky Behavior*			
No	1		1
Some	2.16 (1.74–2.67)		2.09 (1.69–2.49)
High	1.27 (0.88–1.84)		1.28 (0.87–1.85)
*Residence*			
Urban	1	1	1
Rural	0.20 (0.17–0.24)	0.68 (0.54–0.85)	0.96 (0.74–1.26)
*Distance from health facility*			
Big problem	1		1
Not a big problem	1.30 (1.19–1.43)	1.16 (1.06–1.27)	1.08 (0.98–1.16)
*Community wealth index*			
Low	1	1	1
High	2.04 (1.82–2.27)	1.33 (1.03–1.72)	1.20 (1.03–1.57)
*Community-level literacy*			
Low	1	1	1
High	4.65 (3.89–5.55)	1.46 (1.14–1.86)	1.16 (1.05–1.51)
*Community knowledge*			
Low	1	1	1
High	4.79 (4.02–5.70)	2.20 (1.84–2.63)	2.03 (1.68–2.45)
*Community stigma*			
Low	1	1	1
High	0.61 (0.51–0.73)	0.59 (0.50–0.69)	0.89 (0.76–1.04)
*Community sexual risk*			
Low	1	1	1
High	2.11 (1.71–2.59)	1.17 (1.01–1.36)	1.04 (0.89–1.23)

## Discussion

In the development of the current HIV National Strategic Plan, the government of Ethiopia has adopted the global goal to attain the 90–90-90 targets through intensify targeted HIV testing and counseling services, attain virtual elimination of MTCT, optimize and sustain quality care and treatment [16]. This study uses a nationally representative cross-sectional sample of women to investigate the individual and community-level determinants that affect the acceptance of VCT among women of reproductive-age in Ethiopia. Therefore, the results of this study indicate that several individual and community-level determinants are related to women of childbearing age receiving VCT.

In this study, the VCT uptake among ever-married women was high compared to never-married women. This finding is consistent with other studies conducted in Ethiopia [18, 25]. The high rate of testing among married women might be due to the majority of women believe that VCT is useful for preparing for marriage [20], and following marriage women are more likely to visit the health facility for perinatal service available in most health institutions and so for VCT.

The probability of having been tested was highest among women aged 35 years or older and 25–34 years. The results of this survey are consistent with those of other studies, indicating that the VCT uptake varies with age [18, 19, 22, 23, 25, 26, 28]. Previous documented study on awareness and knowledge about HIV/AIDS among women of reproductive age indicated that the odds of HIV/AIDS awareness and knowledge increase with women’s age, which might increase VCT uptake [31]. This fact is likely because the fear of stigma and discrimination from the society towards VCT uptake were less common among the older age compared to younger age group [28].

We found that VCT uptake increased with educational level and family wealth. This finding relates to other studies elsewhere in which women with a higher education level [1, 20–24] and higher family wealth [18, 19, 22–24] have a higher chance of HIV testing. This result highlighted the importance of education and higher wealth to the increment of HIV testing and counselling. The possible reasons may be the increased awareness and knowledge of HIV among educated women and women from the wealthiest households [31]. Also, this association was likely due to women with higher income and educational level were more likely to seek maternal health care services, have women’s autonomy, and they are near to information [32, 33]. Another possible justification might be that in this study, most women from the wealthiest families (91%) and higher education levels (94%) had ≥ 4 ANC follow-ups, which may increase VCT. However, the current study disagrees with a study conducted in China, which shows a negative association between income and VCT uptake [27]. This disagreement might be due to the differences of tools used for wealth index measurement, in which DHS program used Principal Component Analysis to calculate wealth index (a composite measure of a household's cumulative living standards), while the previous study used monthly income.

As previously documented, we observed that women with comprehensive HIV-related knowledge are more likely to receive VCT [18, 21, 23–26, 28, 34]. A possible explanation may be that knowledgeable women may be aware of the benefits of getting an HIV test. This finding suggests that discussing HIV will increases the acceptance of HIV testing; therefore, dialogue on this issue and prevention of stigma are essential [35, 36].

The uptake of VCT was higher among individuals with risky sexual behavior. This was consistent with studies conducted in different countries [18, 19, 22, 24, 26]. This is because women with risky sexual behaviors are afraid and uncertain about their sero-status, which will cause them to be tested for HIV than those without risky sexual behaviors.

In agreement with previous studies conducted in Ethiopia [18, 19, 22], Nigeria [23], Malawi [21] and China [27], this study found that women who have a stigma against PLWHA had a decreased VCT uptake. This association could be possibly due to the cultural and moral values attached to sex orientation that greatly determine people’s attitude towards PLWHA. Individuals who are infected with HIV are perceived to be engaged in socially disapproved pre-marital or extra-marital sexual affairs, that might cause misconception of HIV testing due to the fear of negative consequences of the social disapproval [37].

This study also assesses the association between community-level determinants and VCT uptake. Our result indicated that VCT uptake was higher among women living in communities where the proportion of respondents were more educated than the median and where women were from wealthier communities, which was in agreement with the findings from a study conducted in Burkina Faso [24]. Also, this study suggested that women living in communities with high knowledge related to HIV were more like to be tested for HIV, i.e. living in communities where HIV is actively addressed seems to have a strong effect on the willingness to get the test. This is similar to previous literature, which indicates individuals’ engagement in community group discussions about HIV had increased odds of VCT uptake compared to those who had not participated [36]. A study showed that community characteristics/interventions are very effective in increase the use of preventive measures [35].

### Strength and limitation of the study

The main strength of this study is the use of nationally representative data, which was collected using standard and validated data collection tools. In addition, a high-level model (multilevel analysis) was used, which takes into account the relevance of EDHS data when determining estimates. However, our study is without limitations. Due to the secondary nature of the data, Factors such as availability of treatment, health professional related factors, and support programs were not included in the analysis.

## Conclusion

This study revealed that not only individual level factors but also community level factors determine the status of HIV voluntary counseling and testing. Amongst individual level determinants: having primary, secondary, and higher education, older age, being married, being wealthier, having comprehensive knowledge and having risky sexual behavior were positively associated with VCT uptake, but having stigma was negatively associated with VCT uptake. The community-level determinants associated with VCT uptake were community wealth, community-level literacy and community knowledge. Hence, efforts must be made to address the factors that hinder VCT uptake to improve VCT uptake among women in order to control the spread of HIV/AIDS. Therefore, any stakeholder engaged in HIV/AIDS prevention and control in the country should consider factors such as educational levels and wealth status at both individual and community-levels that shape uptake of VCT in Ethiopia. Moreover, there should be an integrated intervention to scale up HIV knowledge through community-based educations, which in turn help to avoid risky sexual behavior and stigma to improve VCT among women to meet the ambitious plan of ending HIV as a public health threat by 2030.

## Data Availability

The dataset can be obtained from the online request of DHS Program (http://www.dhsprogram.com). Minimal data can also be provided through contacting the corresponding author on reasonable request.

## Abbreviations

AOR: Adjusted odds ratio
CI: Confidence interval
EDHS: Ethiopian Demographic and Health Survey
HIV: Human immunodeficiency virus
LLR: Log likelihood ratio
PLWHA: People living with HIV
VCT: Voluntary counseling and testing
